# Complete mitochondrial genome of the *Cyclemys pulchristriata* (Chelonia: Geoemydidae)

**DOI:** 10.1080/23802359.2017.1347836

**Published:** 2017-07-11

**Authors:** Jun Li, Yuanhua Lu, Jiawei Zan, Liuwang Nie

**Affiliations:** a College of Life Science, Anhui Normal University, Wuhu, China;; b The Laboratory of Conservation and Exploitation Research of Biological Resources in Anhui, Wuhu, Anhui, China

**Keywords:** *Cyclemys pulchristriata*, mitochondrial genome, phylogenetic evolution

## Abstract

In this study, we obtained complete mitochondrial genome sequence of *Cyclemys pulchristriata*. The mitochondrial genome reaches a length of 16,527 bp, containing 13 protein-coding genes (PCGs), 22tRNA genes, 2 rRNA genes and 1 control region. All protein-coding genes initiate with ATG as start codon, except for *CO1* started with GTG. Most protein-coding genes ended by TAA as stop codon. Interestingly, there is an extra nucleotide A insertion in *ND3* gene in *C. pulchristriata*. This study provides information on the genetic resources of *C. pulchristriata* that will contribute to protect this species.

*Cyclemys pulchristriata* distributed in the area of central Vietnam and eastern Kampuchea (Couture 1990). The species in *Cyclemys* are similar in size and form. It can distinguished from other congeneric species in the details of dorsal and abdomen (Fritz et al. 2008). As the species with a small population quantity, clarifying the specific genetic information, is an important requirement to formulate effective protective measures, *C. pulchristriata* mitochondrial genome sequence has not been reported.

In this, we sequenced and characterized the complete mitochondrial genome of *C. pulchristriat*a. A sample was collected at Shanghai Zoo and stored in the Provincial Key Lab of the Conservation and Exploitation Research of Biological Resources in Anhui Normal University. Total genomic DNA was extracted from muscle samples by the standard phenol–chloroform method (Kan et al. 2010). Mitochondrial genome was amplified with 16 primers using PCR and then sequenced. These primers were designed by Oligo 7.0 based on the complete mitochondrial genome of *C. atripons* (GenBank: EF067858). BioEdit 7.2.3 was used to assist artificial sequence splicing after sequencing (Zhou et al. 2015). The mitochondrial genome sequence of *C. pulchristriata* was submitted to GenBank for accession number NC_026027.

Complete mitochondrial genome sequence of *C. pulchristriata* has a circular genome of 16,527 bp, containing 13 protein-coding genes, 22 tRNA genes, 2 rRNA genes and 1 control region. The overall base composition of mitogenome was A(34.4%), T(27.2%), C(25.4%) and G(13.0%), respectively. All protein-coding genes initiate with ATG as start codon, except for *CO1* started with GTG. Most protein-coding genes ended by TAA as stop codon. The lengths of 12S ribosomal RNA and 16S ribosomal RNA are 965 bp and 1597 bp, respectively. The length of control region is 1149 bp, ranging from 15,513 to 16,527 bp. Interestingly, there is an extra nucleotide A insertion in *ND3* gene in *C. pulchristriata*.

The phylogenetic trees of *C. pulchristriata* were determined by the nucleotide sequence of the 13 PCGs using neighbour-joining (NJ)/maximum parsimony (MP)/maximum-likelihood (ML) analyses (Figure 1). A total of 26 complete mitochondrial genomes were sampled for phylogenetic analysis. Mega 6.06 was used for NJ analyses and PAUP 4.0 beta 10 was used for MP analyses (Swofford 2002). The NJ analyses were conducted using 1000 bootstrap and MP analyses were performed with 1000 bootstrap values. ML analysis was performed in RAxML GUI v 1.3.1 under GTRGAMMAX model, which was implemented in 1000 bootstrap values (Lan et al. 2015). There are 24 species from the Geoemydidae, which contain *Batagur*, *Cuora*, *Cyclemys*, *Heosemys*, *Mauremys*, *Notochelys* and *Sacalia*, using *Notochelys platynota* and *Testudo kleinmanni* as outgroup.

**Figure 1.**
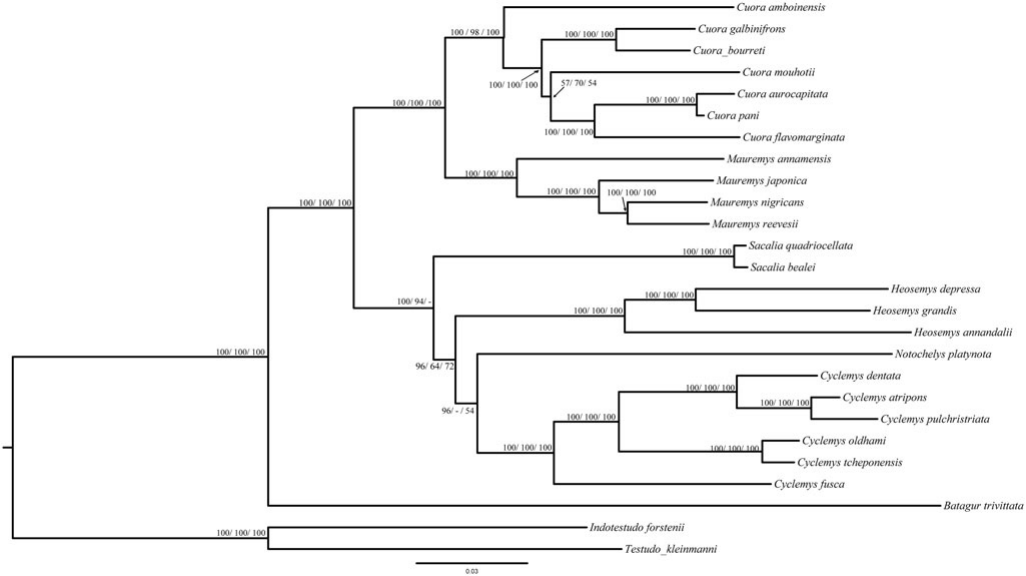
Phylogenetic tree of *C. pulchristriata* based on the nucleotide dataset of the 13 PCGs. *Notochelys platynota* and *Testudo kleinmanni* as outgroup. Number above each node indicates the ML/MP/NJ bootstrap support values. All 26 species accession numbers are listed as below: *Batagur trivittata* NC_032300, *Cuora amboinensis* NC_014769, *Cuora aurocapitata* NC_009509, *Cuora bourreti* NC_017885, *Cuora flavomarginata* NC_012054, *Cuora galbinifrons* NC_014102, *Cuora mouhotii* NC_010973, *Cuora pani* NC_014401, *Cyclemys atripons* NC_010970, *Cyclemys dentata* NC_018793, *Cyclemys fusca* JX218031, *Cyclemys oldhamii* NC_023220, *Cyclemys pulchristriata* NC_026027, *Cyclemys tcheponensis* NC_023221, *Heosemys annandalii* NC_020668, *Heosemys depressa* NC_026024, *Heosemys grandis* NC_032297, *Indotestudo forstenii* NC_007696, *Mauremys annamensis* NC_017875, *Mauremys japonica* NC_016951, *Mauremys nigricans* NC_029369, *Mauremys reevesii* KJ700438, *Notochelys platynota* NC_020665, *Sacalia bealei* NC_016691, *Sacalia quadriocellata* NC_011819 and *Testudo kleinmanni* NC_007699.

According to clusters of the phylogenetic trees, the evolutionary relationships between *C. atripons* and *C. pulchristriata* are most close. This conclusion is consistent with previously reported results (Stuart and Fritz 2008). *Cyclemys* initially clustered with the *Notochelys*, followed by clustering with the *Heosemys.* Twenty-four representative turtles of *Geoemydidae* were divided into seven families. This study provides information on the genetic resources of *C. pulchristriata* will contribute to protect this species.
